# Complementary and Alternative Medicine Therapies in relation to Self-Reported Better Sleep across Racial and Ethnic Groups in the United States

**DOI:** 10.64898/2026.09.04.26362273

**Published:** 2026-09-08

**Authors:** Rupsha Singh, Symielle A. Gaston, Christopher Payne, Dayna T. Neo, Suzanne M. Bertisch, Wayne B. Jonas, Chandra L. Jackson

## Abstract

**Objectives:**

Complementary and Alternative Medicine (CAM) approaches vary across race and ethnicity, reflecting different cultural practices. However, its relationship with sleep is understudied. We investigated cross-sectional CAM-sleep associations across racial and ethnic groups.

**Methods:**

In the nationally-representative 2012 National Health Interview Survey, participants identified their most important CAM therapies and whether the therapy yielded ‘better sleep’ (yes/no). We used Poisson regression with robust variance to estimate adjusted prevalence ratios (aPRs) and 95% confidence intervals (CIs) and examined effect measure modification using Wald tests of interaction terms.

**Results:**

Among 9,308 adults (mean age±SE = 46.4±0.3 years), 58.2% were women. CAM therapy prevalence ranged from 0.1% (craniosacral therapy) to 28.9% (herbal supplements). The proportion perceiving ‘better sleep’ was 41.3%. When deemed most important for health, massage; meditation; movement or exercise techniques; and yoga, Tai Chi, and Qigong were associated with higher prevalence of perceiving ‘better sleep’ similarly across racial and ethnic groups. Chiropractic or osteopathic manipulation was associated with perceiving better sleep only among non-Hispanic (NH)-White adults (aPR=1.14, 95% CI:1.05-1.24). The highest magnitude of associations between herbal supplement use and lower prevalence of ‘better sleep’ was among NH-White adults (aPR=0.29, 95% CI:0.25-0.34 vs. aPR range:0.37 (NH- Asian) to 0.50 (NH-Black)). Limited by a small sample size, no associations were observed among NH-American Indian/Alaska Native participants.

**Conclusion:**

Many CAM therapies were associated with perceiving better sleep across racial and ethnic groups; however, effectiveness varied by therapy and racial and ethnic groups, yielding mixed evidence of their potential role in sleep health promotion.

## Full Text Availability

The license terms selected by the author(s) for this preprint version do not permit archiving in PMC. The full text is available from the preprint server.

